# Supplementary material for: Machine learning for predicting Chagas disease infection in rural areas of Brazil
Source: PLoS Negl Trop Dis. 2024 Apr 16;18(4):e0012026. doi: 10.1371/journal.pntd.0012026 (PMC11093296; doi:10.1371/journal.pntd.0012026)
Supplement: Attachment — Submitted filename: response_reviewers.docx [file pntd.0012026.s002.docx]

Dear Reviewers,

I trust this message finds you well. I want to express my sincere appreciation for your time and meticulous review of our manuscript." Your insights are invaluable, and we are grateful for the opportunity to address your thoughtful questions and concerns.

Your careful scrutiny has provided us with valuable perspectives, and we are dedicated to ensuring that our work meets the high standards set by PLOS Neglected Diseases. Below, I address each of your questions with the aim of providing clarity and addressing the gaps you have identified:

**Methods:**

**Reviewer** #1: The study's objectives are clearly outlined, but there is a discrepancy in the datasets mentioned and used by the authors. Initially, a database consisting of 500 patients who tested positive for Chagas Disease, 500 who tested negative, and an additional 100 diagnosed with cardiomyopathy is introduced. However, the dataset actually employed in this study includes a different patient set: 196 tested positive for Chagas Disease and 1,810 tested negative.

***Answer:*** *Initially, we used a dataset comprising 500 patients who tested positive for Chagas Disease, 500 who tested negative, and an additional 100 diagnosed with cardiomyopathy. This dataset was derived from a previous study and (as it contained a very detailed questionnaire) we utilized this initial dataset to create a targeted questionnaire, focusing on questions with a higher likelihood of identifying patients with Chagas Disease. This strategic refinement aimed to optimize the identification process for individuals at risk. Subsequently, we applied this questionnaire in a real-life scenario, conducting a comprehensive search for patients with Chagas Disease in Minas Gerais. In this subsequent phase, we identified 196 patients who tested positive for Chagas Disease and 1,810 who tested negative.*

**Reviewer**: There also appears to be a discrepancy regarding the study's location. Reference (6) mentions blood donors from the state of São Paulo, while this study was conducted in two municipalities in Minas Gerais. It is crucial to clarify which dataset was actually used.
***Answer:***  *Regarding the study location, I would like to clarify that the questionnaire development initially were from blood donors from the state of São Paulo. Subsequently, the questionnaire was validated in two municipalities in Minas Gerais, and it is this dataset that was used for training the Machine Learning algorithm. We acknowledge the importance of explicitly stating this in the manuscript to avoid any ambiguity, and we will ensure this clarification is incorporated in the revised version.*

**Reviewer**: The authors of the study used the ELISA test as a diagnostic criterion. However, according to the II Brazilian Consensus on Chagas Disease (1), the confirmation of Chronic or Acute Chagas Disease is determined by the reactivity of anti-T. cruzi (IgG) serology, which should be assessed using two distinct methods: ELISA, HAI, or IFI. The Clinical Protocol and Therapeutic Guidelines for Chagas Disease (2) further specify that for the chronic phase of the disease, a negative result from a single rapid test rules out the disease. Conversely, a positive test necessitates diagnostic confirmation by one of the following tests: ELISA, IFI, HAI, WB, or CLIA.
In this study, however, patient testing was conducted solely using the ELISA method. The exclusive reliance on a single test could potentially skew the results, leading to the misclassification of patients without Chagas Disease as positive. This could subsequently impact the accuracy of the model, particularly in relation to positive outcomes.

***Answer:***  *In response to your second point regarding the diagnostic criteria, we conducted additional testing on the samples at the IMT – USP Laboratory using Other ELISA (from diferente manufactors) so wach test was analysed at least twice (by two diferente ELISAs)., positive = 2 different ELISA positives, negative= 2 different Elisa negatives, indeterminated = 1 Elisa positive and 1 Elisa negative*

*These additional tests provide a more comprehensive evaluation of the samples and contribute to the overall robustness of our findings. We acknowledge the importance of adhering to established diagnostic protocols, and we are committed to presenting a more nuanced and accurate representation of our methodology in the revised manuscript.*

Reviewer #2: The file does not explicitly state a testable hypothesis, but it does clearly articulate the objectives of the study, which are to explore the potential of machine learning algorithms to predict Chagas disease risk in rural areas of Brazil.
2. The study design is appropriate to address the stated objectives, as the researchers collected data from a rural population in Brazil and used machine learning algorithms to analyze the data and predict Chagas disease risk.
3. The population is clearly described as rural residents of Brazil, and the study focuses specifically on risk factors related to vectorial transmission of Chagas disease. The population is appropriate for the hypothesis being tested, as the study aims to identify individuals at risk of Chagas disease in rural areas.
Reviewers asked the questions number 4 and 5

4. The file does not provide specific information on the sample size used in the study, so it is unclear whether the sample size is sufficient to ensure adequate power to address the hypothesis being tested.

5. The file does not provide detailed information on the statistical analysis used to support conclusions, but it does describe the machine learning algorithms used to analyze the data and predict Chagas disease risk.

6. The PDF file does not raise any concerns about ethical or regulatory requirements not being met.

**Answer:** I acknowledge the absence of specific information on the sample size in the provided file. In the context of machine learning and predictive models, our methodology differs from traditional hypothesis testing. Rather than relying on a predefined sample size for hypothesis testing, our approach involves utilizing diverse algorithms to predict serological status. We understand the importance of statistical power and will ensure to include relevant information in the revised manuscript to clarify the adequacy of our sample size for the predictive modeling undertaken.

The file indeed lacks detailed information on the statistical analysis supporting our conclusions. In the realm of machine learning, our emphasis lies on the application of various algorithms to analyze and predict Chagas disease risk. We appreciate your recognition of our use of machine learning techniques. To address this concern, we will provide a more comprehensive description of the machine learning algorithms employed and their respective roles in predicting the outcome. This addition aims to offer a clearer understanding of the analytical process and how it supports our conclusions.

**Reviewer #3:** -Are the objectives of the study clearly articulated with a clear testable hypothesis stated?
Yes.
-Is the study design appropriate to address the stated objectives?
Yes.
-Is the population clearly described and appropriate for the hypothesis being tested?
Not completely. The population refers to a previous study )REDS Retrovirus Epidemiology Donor Study, Sabino et al;2013) but no information about symptoms or medical data are given. This may be this may be masking important sampling selectivity.

***Answer:*** *Allow me to clarify that this population (REDS Retrovirus Epidemiology Donor Study) was specifically utilized for the development of a questionnaire aimed at identifying positive serological cases of Chagas disease. The focus of our inquiry was on epidemiological aspects and exposures, and therefore, no symptoms or clinical signs were addressed in the questionnaire. This deliberate omission was intended to streamline the selection of questions related to a positive serological status.*

*The primary goal of our study was to refine the identification of individuals at risk of Chagas disease based on serological markers, utilizing a targeted questionnaire derived from the REDS study. While we acknowledge that this sampling approach may not capture the full clinical spectrum of Chagas disease, it was a deliberate choice to optimize the questionnaire for our specific research objectives.*

**Reviewer**: Is the sample size sufficient to ensure adequate power to address the hypothesis being tested?

***Answer****: In the context of machine learning and predictive models, our methodology differs from traditional hypothesis testing. Rather than relying on a predefined sample size for hypothesis testing, our approach involves utilizing diverse algorithms to predict serological status.

-Were correct statistical analysis used to support conclusions?*

*In the realm of machine learning, our emphasis lies on the application of various algorithms to analyze and predict Chagas disease risk. We appreciate your recognition of our use of machine learning techniques. To address this concern, we will provide a more comprehensive description of the machine learning algorithms employed and their respective roles in analyzing the data. This addition aims to offer a clearer understanding of the analytical process and how it supports our conclusions.*

**Reviewer**: Are there concerns about ethical or regulatory requirements being met?
Yes.

**Answer** : We would like to address them if you could be more specific

**Results:**

Reviewer #1: The results presented are in line with what the authors proposed, and were presented in a clear and objective manner with figures that aid in understanding the article.

Reviewer #2: 1. The file does not provide a detailed analysis plan, so it is unclear whether the analysis presented matches the analysis plan. However, the file does describe the machine learning algorithms used to analyze the data and predict Chagas disease risk, so it is likely that the analysis presented is consistent with the methods used.

2. The results are clearly and completely presented in the file, with detailed information on the performance of the machine learning algorithms and the risk factors associated with Chagas disease.
3. The figures (tables, images) in the PDF file are of sufficient quality for clarity, with clear labels and legends to help readers interpret the data.

Reviewer #3:
-Does the analysis presented match the analysis plan?
somehow yes

-Are the results clearly and completely presented?
yes

-Are the figures (Tables, Images) of sufficient quality for clarity?
yes.

**Conclusions:**

**Reviewer #1**: The conclusions drawn in the study align with its findings. However, it is crucial for the authors to discuss the potential limitations of applying the model to the Unified Health System (SUS), given that only one serological technique was used.

The failure to perform two tests, as recommended by testing guidelines, could potentially limit the model’s applicability to the SUS. This is primarily due to the model’s potential to overestimate the number of positive cases, given its reliance on a single serological test.

***Answer:*** *We fully recognize that testing guidelines in Brazil recommend the combination of two different methodologies to provide a diagnostic for Chagas disease. In our study, we aimed to address this by combining two high-sensitivity ELISAs from different manufacturers. This deliberate approach was undertaken to enhance sensitivity and, in turn, improve the accuracy of our model.*

Reviewer #2: 1. The conclusions presented in the PDF file are supported by the data presented, with detailed information on the performance of the machine learning algorithms and the risk factors associated with Chagas disease.
2. The limitations of the analysis are clearly described in the PDF file, including concerns about the small sample size and the specificity of the results for the population in question. The authors also note that the same questions may not perform uniformly across different regions, and that environmental conditions and the presence of other types of vectors can impact vectorial capacity.
3. The authors do discuss how these data can be helpful to advance our understanding of the topic under study, noting that machine learning algorithms can be a useful tool in diagnosing patients with Chagas disease and prioritizing referrals for serology testing based solely on questionnaire data. They also suggest that future studies should consider environmental factors and other types of vectors to develop more effective prevention and control measures.
4. The public health relevance of the study is addressed in the PDF file, with a focus on the potential of machine learning algorithms to improve screening and early detection of Chagas disease in rural areas of Brazil. The authors note that Chagas disease is a significant public health concern in Latin America, and that early detection and treatment can help prevent the spread of the disease.

**Reviewer #3:** -Are the conclusions supported by the data presented?
No. In my opinion, the conclusion about saving testing resources is not correct. I clarify this below.
***Answer:*** *We agree that the saving test resources is not the focal point of our analysis, and we now recognize the complexity of conducting an economical evaluation of Chagas disease diagnostics, especially considering the intricate landscape of the Brazilian healthcare system and the challenges in providing care to Chagas disease patients. Our revised discussion places greater emphasis on the nuanced understanding of these complexities.*

*At the same time, we maintain our belief that a tool designed to identify individuals at higher risk could be valuable, particularly in scenarios where resources for testing are limited. Our intention is to highlight the potential utility of such a tool, even within the challenging context of resource constraints.*

-Are the limitations of analysis clearly described?
yes.
-Do the authors discuss how these data can be helpful to advance our understanding of the topic under study?
Somehow.
-Is public health relevance addressed?
Yes.

Thank you once again for your time and insightful feedback. Your expertise and constructive comments have been invaluable in refining our manuscript. We are committed to addressing all your suggestions and believe that your guidance will significantly enhance the quality and impact of our work.

We look forward to the opportunity to contribute to the scientific discourse in PLOS Neglected Diseases, and we are open to any further suggestions or queries you may have. Your dedication to advancing scientific knowledge is deeply appreciated.

Best regards,

Fabio De Rose Ghilardi

University of São Paulo, Brazil
